# Supplementary figures and images for: Genomic Diversity across Candida auris Clinical Isolates Shapes Rapid Development of Antifungal Resistance In Vitro and In Vivo
Source: mBio. 2022 Jul 5;13(4):e00842-22. doi: 10.1128/mbio.00842-22 (PMC9426540; doi:10.1128/mbio.00842-22)

**A**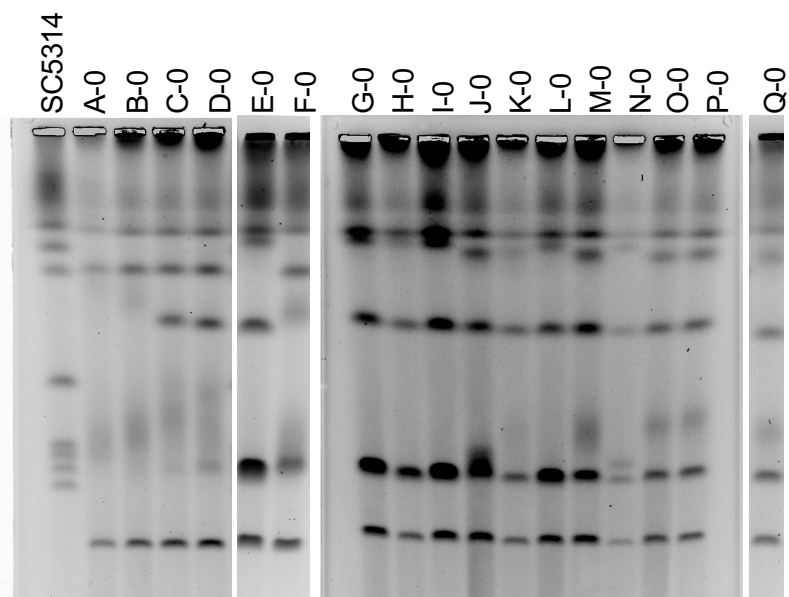**B**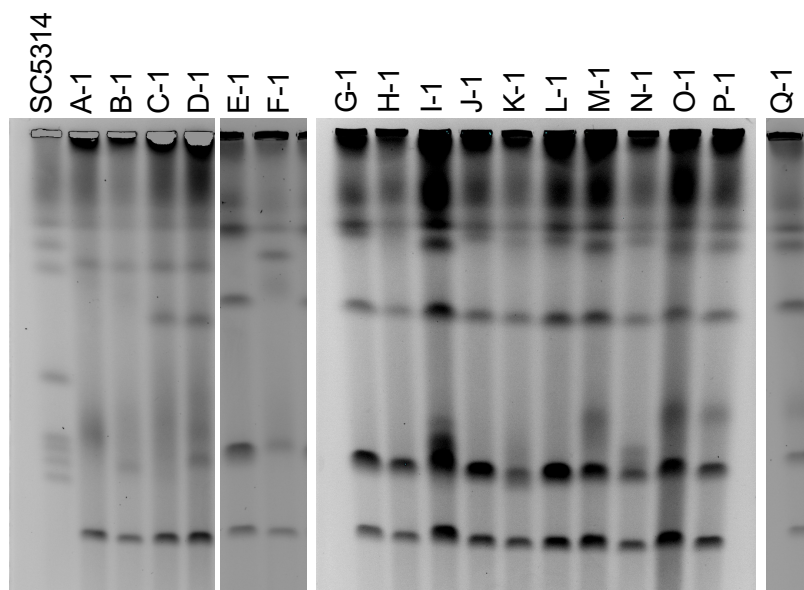**C**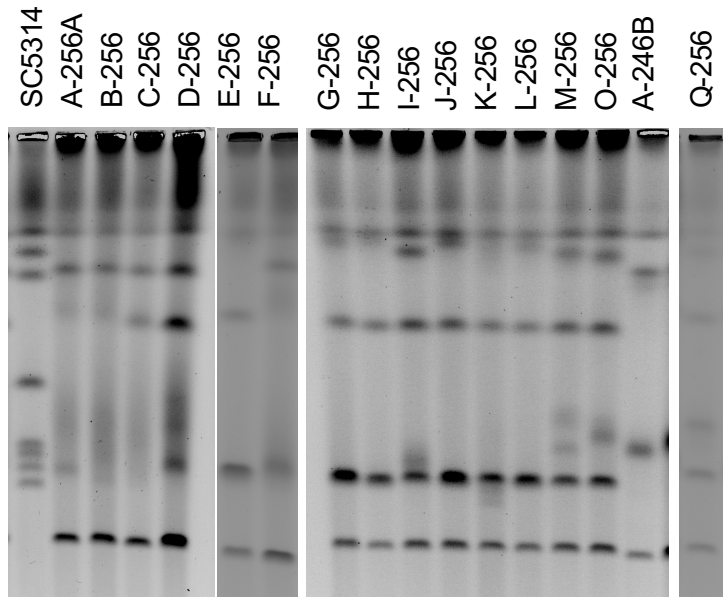

Supplement: FIG S2 [file mbio.00842-22-s0007.pdf]

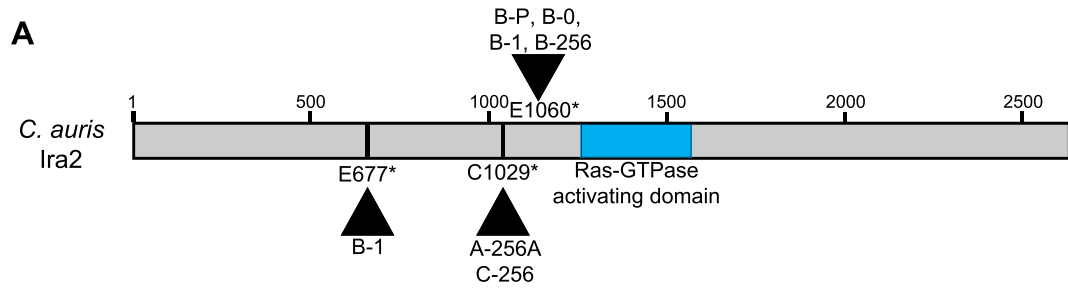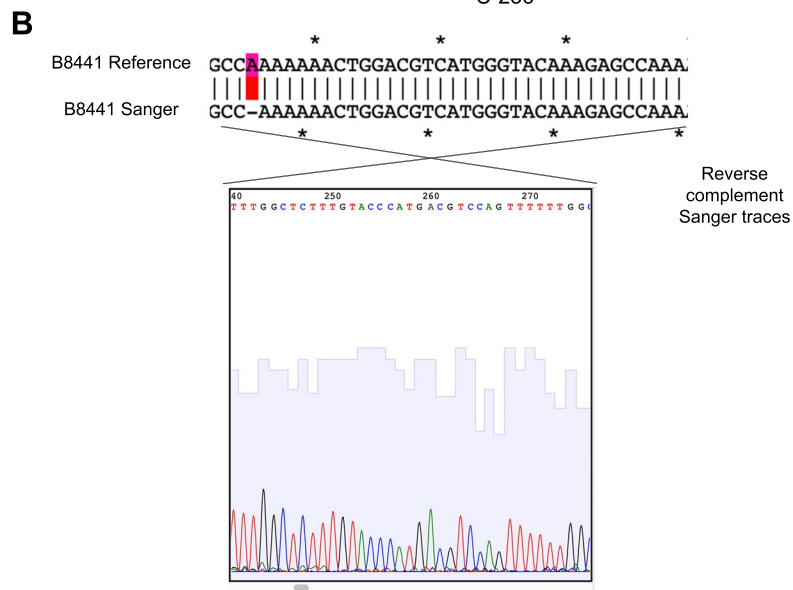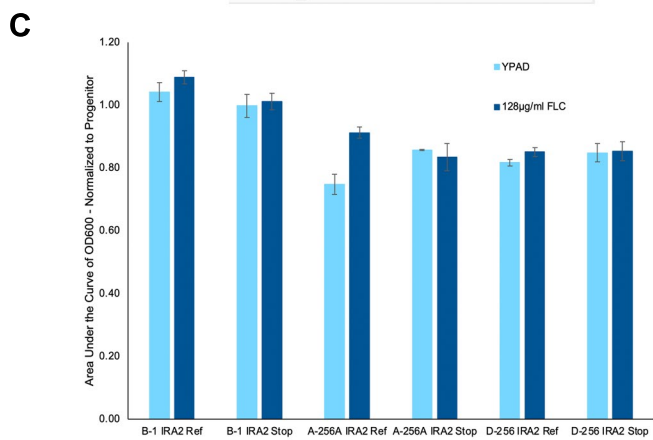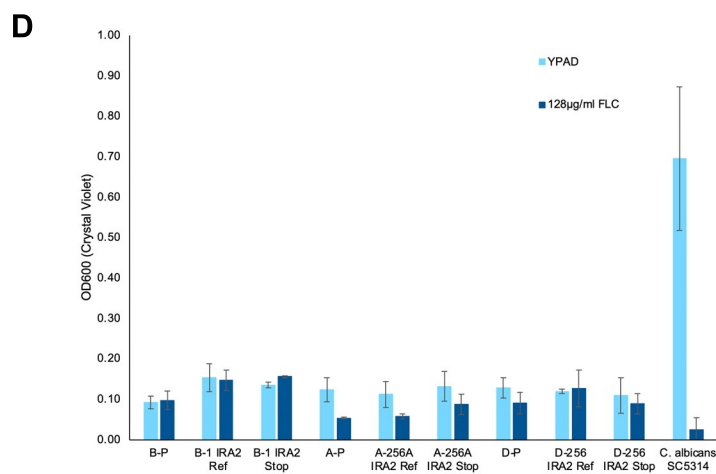

Supplement: FIG S3 [file mbio.00842-22-s0008.pdf]

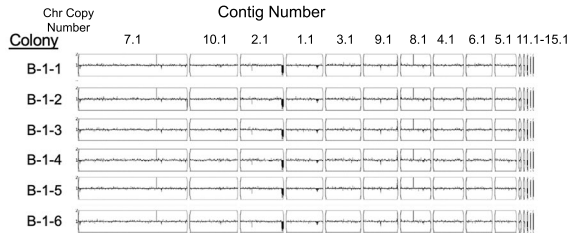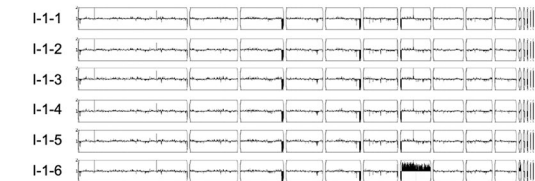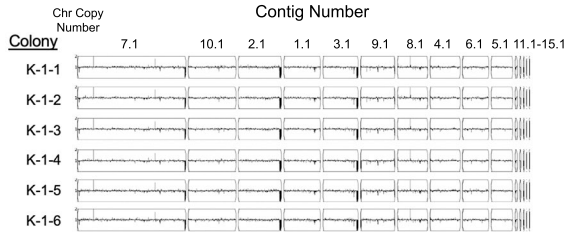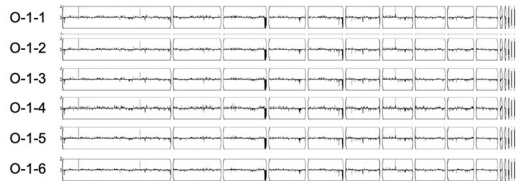

Supplement: FIG S4 [file mbio.00842-22-s0009.pdf]
