# Supplementary material for: Genomic Diversity across Candida auris Clinical Isolates Shapes Rapid Development of Antifungal Resistance In Vitro and In Vivo
Source: mBio. 2022 Jul 5;13(4):e00842-22. doi: 10.1128/mbio.00842-22 (PMC9426540; doi:10.1128/mbio.00842-22)
Supplement: FIG S1 [file mbio.00842-22-s0006.pdf]

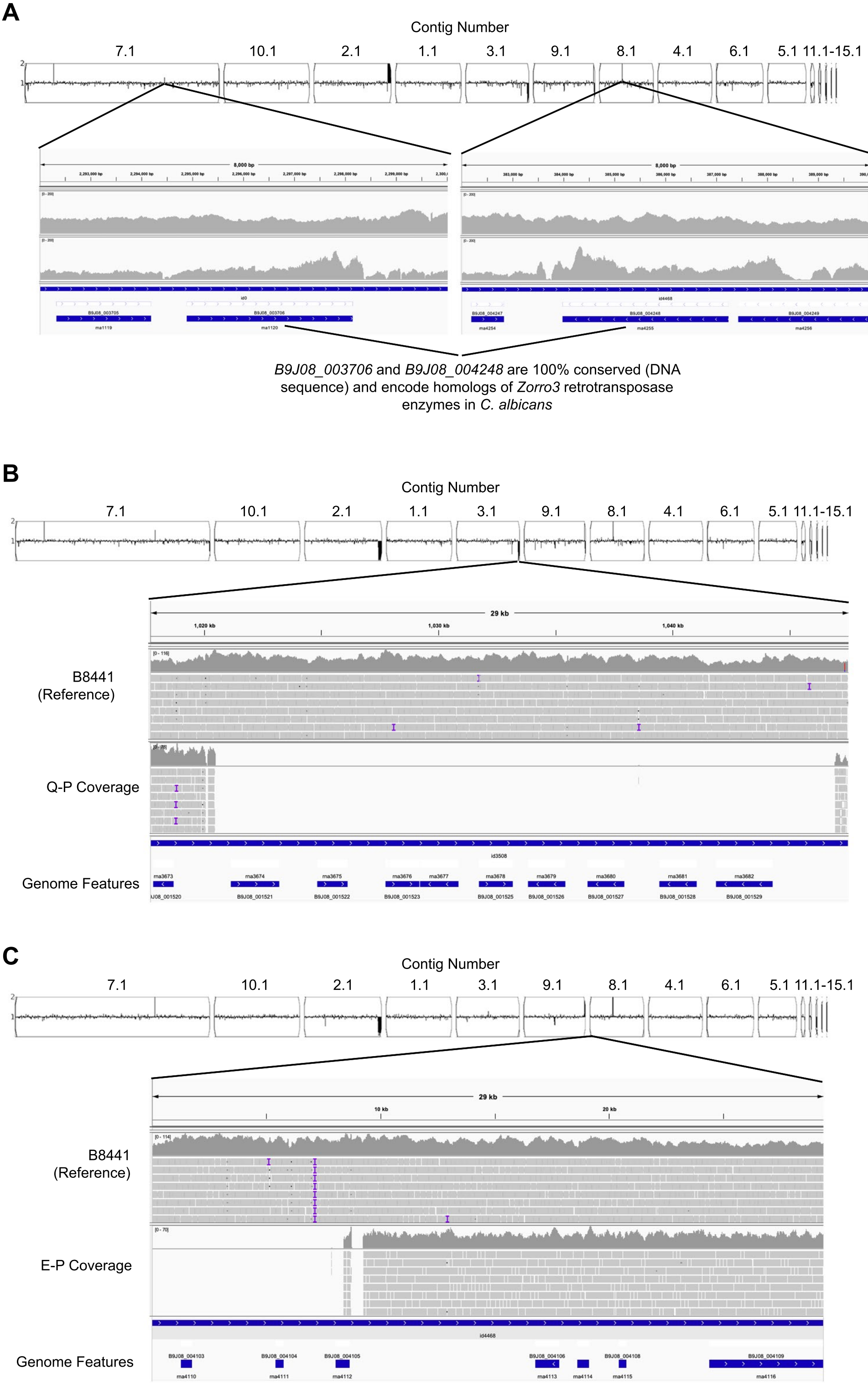
A
Contig Number
7.1
10.1
2.1
1.1
3.1
9.1
8.1
4.1
6.1
5.1 11.1-15.1
8,000 bp
8,000 bp
B9J08_003705
B9J08_003706
B9J08_004247
B9J08_004248
B9J08_004249
B9J08_003706 and *B9J08_004248* are 100% conserved (DNA sequence) and encode homologs of *Zorro3* retrotransposase enzymes in *C. albicans*
B
Contig Number
7.1
10.1
2.1
1.1
3.1
9.1
8.1
4.1
6.1
5.1 11.1-15.1
29 kb
1,020 kb
1,030 kb
1,040 kb
B8441
(Reference)
Q-P Coverage
Genome Features
C
Contig Number
7.1
10.1
2.1
1.1
3.1
9.1
8.1
4.1
6.1
5.1 11.1-15.1
29 kb
10 kb
20 kb
B8441
(Reference)
E-P Coverage
Genome Features
B9J08_004103
B9J08_004104
B9J08_004105
B9J08_004106
B9J08_004108
B9J08_004109
